# The role of place-based policies on carbon emission: A quasi-natural experiment from China’s old revolutionary development program

**DOI:** 10.1016/j.heliyon.2023.e15964

**Published:** 2023-04-29

**Authors:** Dan Pan, Yiqun Chen, Fanbin Kong

**Affiliations:** a School of Economics, Jiangxi University of Finance and Economics, Nanchang, Jiangxi, 330013, China; b School of Economics and Management, Zhejiang A&F University, Hangzhou, Zhejiang, 311330, China; c Institute of Ecological Civilization & Institute of Carbon Neutrality, Zhejiang A&F University, Hangzhou, Zhejiang, 311330, China; d School of Economic Management, Nanjing Forestry University, Nanjing, Jiangsu, 210037, China

**Keywords:** Place-based policies, Carbon emission, China’s old revolutionary development program, Difference-in-difference method

## Abstract

The effectiveness of place-based policies on carbon emission is controversial, and particularly the mechanism behind its effectiveness is unknown. We treat China’s Old Revolutionary Development Program (ORDP)— a large-scale and novel type of place-based policy targeted at undeveloped regions, as a natural experiment to estimate ORDP’s impact on carbon emission. Employing the panel data of 110 prefecture-level cities in China from 2010 to 2019, we perform a time-varying difference-in-differences (DID) study and discover that ORDP leads to an average of 26.7% increase in carbon emission and this effect takes a period to emerge and is not sustainable in the long term. Three mechanisms that may result in such impact are that ORDP improves economic development, changes industrial structure, and decreases technological progress. Further heterogeneity analysis indicates that ORDP results in a greater increased impact on carbon emission in old revolutionary cities that are located in western China compared to those located in central and eastern China.

## 1 Introduction

Increasing carbon emission has become an extraordinarily serious global challenge, especially in economically developing countries due to their exponential economic growth. It is reported that China was responsible for 27% of the world’s total carbon emission in 2019 [[1], [2], [3]], exceeding the total emission of the Organization for Economic Cooperation and Development Countries [4]. This massive carbon emission has caused great harm to the environment and people’s welfare and is a major threat to the accomplishment of the 2030 Sustainable Development Goals. The Chinese government, which has been under intense pressure to reduce carbon emission, has announced numerous policies since 2005, promising to lower carbon intensity by 40%–45% compared to the level in 2005, reaching the peak of carbon emissions in 2030, and finally accomplishing the aim of carbon neutrality in 2060 [1,5,6].

Numerous studies have investigated how to reduce carbon emission and have confirmed that governmental policies play a vital role in controlling carbon emission. For example, existing related works of literature have found that China’s Western Development Strategy [7], China’s WTO accession [8], and China’s pilot low-carbon city policy have a positive effect on carbon emission reduction [9]. The expansionary commercial policies in Australia and the consumer-oriented policies in European countries are also discovered to have a considerable long-term positive effect on carbon emission reduction [10,11]. However, as far as we are aware, little relevant literature has empirically investigated the impacts of place-based policies on carbon emission reduction.

In reality, place-based policies, namely spatially targeted development programs, may serve an important role in controlling carbon emission. On the one hand, place-based policies will decrease carbon emission since the measures implemented in place-based policies can optimize resource allocation, facilitate technological progress, and improve resource utilization efficiency [12]. On the other hand, place-based policies may increase carbon emission since that place-based policies can increase production capacity by enlarging production scale and often result in industry agglomerations, which may lead to accelerated pollution emissions [7]. Hence, the effect of place-based policies on carbon emission has not been confirmed and the underlying linkages between the above two contradictory effects deserve attention. Therefore, we present the following research questions: what is the role of place-based policies on carbon emission? What are the impact mechanisms? Do place-based policies have a heterogeneous effect on carbon emission?

**Fig. 1:**
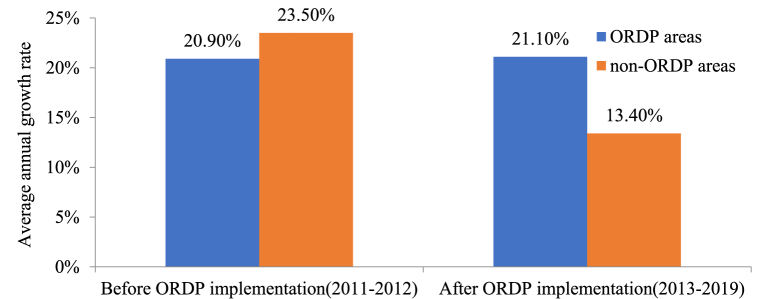
The average annual growth rate of carbon emission of ORDP and non-ORDP areas before and after the implementation of ORDP.

China’s Old Revolutionary Development Program policy (ORDP), a large-scale and novel type of place-based policy introduced by China in 2012, is employed as a quasi-natural experiment of place-based policy to examine the effect of place-based policy on carbon emission. Since 2012, China has launched the ORDP which aims at economic growth and poverty alleviation in five major revolutionary old areas. To obtain the extensive objectives outlined in ORDP, governments have issued a series of preferential policies in finance, land, talent, and industry to support the development of old revolutionary base areas. It is reported that during the “13th Five-Year Plan” period, one out of eight Chinese central budget funds were used in these five major old revolutionary regions, with the investment scale exceeding RMB 300 billion. With these massive preferential investments, the economic growth of the ORDP areas has been considerably boosted. From 2012 to 2019, the average annual growth rate of gross domestic product (GDP) in ORDP areas is 11.7%, while this number is only 9.9% in non-ORDP areas [13]. However, accompanied by the fast economic growth brought by ORDP implementation, the carbon emission in ORDP areas due to ORDP enforcement has also increased. As Fig. 1 shows, before the implementation of ORDP in 2012, the average annual growth rate of carbon emission in ORDP areas is 20.9%, which is lower than the 23.5% annual growth rate in non-ORDP areas. However, after the implementation of ORDP in 2012, the total carbon emission in ORDP areas grew from 52.3 million tons in 2012 to 244.2 million tons in 2019, at an annual growth rate of 21.1%, which is considerably higher than the annual growth rate of 13.4% in non-ORDP areas, indicating that ORDP may be a contributor to the growth of carbon emission in ORDP areas. Nevertheless, few studies have been dedicated to empirically examining the impact of ORDP on carbon emission. We focus on answering the following three questions: (1) Do ORDP cities and non-ORDP cities show differences in the level of carbon emission? (2) Is there a significant change in carbon emission in ORDP cities after ORDP implementation? (3) What is the mechanism of ORDP’s effects on carbon emission? The answers to these questions may be of great significance for the ORDP areas to achieve the dual goals of economic growth and carbon emission reduction and thus provide a “feasible plan” to find the balance between economic prosperity and environmental protection for China.

Our paper makes contributions to four strands of literature. First, we show the first stringent empirical proof of the impacts of place-based policies on the environment. In terms of sustainable economic, social, and environmental development, place-based policies are essential to supporting governments in providing high-quality living and well-being for their people. Most existing studies have examined how place-based policies affect economic growth [14,15], governance challenges [16], and balanced economic development within and between regions [17]. For example, Jia J. et al. [14] documented that China’s Great Western Development Program has increased the targeted regions' annual GDP growth by 1.6%. Lu Y. et al. [18] found that the Economic Zone Program in China has a beneficial effect on industrial production in the targeted regions. Barbieri E. et al. [19] found that the first special economic zone boosts industrial output and economic development in China. However, few studies are concerned about the effects of place-based policies on the environment, particularly in developing countries, which prevents us from gaining a comprehensive understanding of place-based policies. We provide evidence that place-based policies will lead to an increase in carbon emission. Furthermore, we explore the heterogeneity effect of place-based policies more closely. Our results can offer valuable recommendations to simulate decisions regarding China’s future place-based policies.

Second, in comparison to existing literature that mostly focuses on examining the environmental performance of place-based policies in developed nations, such as America, Australia, and some European countries [20], we examine how the carbon emission in China, the largest developing country in the world, is affected by place-based policies. The environmental performance of place-based policies has enormous disparities between developing and developed countries for the following two reasons. One reason is that the implemented areas of place-based policies in these two systems are different, leading to dissimilar environmental performance. As Zheng S. et al. [21] pointed out that place-based policies are frequently implemented by developed economies in economically depressed areas while developing economies implement them in places with a stronger economic foundation or favorable geographic conditions. Another reason is that place-based policies' impacts on carbon emission in developing nations are more complicated than that in developed countries, so more empirical studies are needed. For one thing, developing economies frequently have various market failures, such as weaknesses in the credit market and inefficient resource allocation, and measures in place-based policies can lessen these market failures [12]. Thus place-based policies will have a greater impact on carbon emission reduction in developing countries than in developed countries. For another thing, place-based policies may be unsuccessful in decreasing carbon emission or even increasing carbon emission in developing economies because of the institutional mechanism weaknesses, corruption, and other problems in these economies [22], The ORDP in our paper is an example of place-based policies targeted at the disadvantaged areas in the biggest developing country in the world—China. Thus, we present a thorough empirical evaluation of how place-based policies may affect carbon emission in the context of disadvantaged areas in developing countries.

Third, we contribute to the burgeoning debate about the relationship between economic development and environmental sustainability. A growing literature has been engaged in figuring out the linkage between economic development and environmental sustainability, but there have been opposite views for a long time [23]. Traditional economics affirmed that there is a trade-off relationship between these two [24], while recent literature asserted that these two can go hand in hand [25]. By using a relatively rigorous empirical strategy, we found that ORDP— a policy aimed at economic growth and poverty alleviation, will lead to an increase in carbon emission by 26.7% in China’s old revolutionary cities, which provides new evidence of the controversial relationship between economic development and environmental sustainability, especially in disadvantaged areas in developing countries.

Fourth, we formulate a theoretical framework and empirically test the mechanisms through which the effectiveness of place-based policies on carbon emission happens. The majority of current research focuses on evaluating the effect of place-based policy [26]. However, there is little discussion on the mechanisms affecting that effect. This paper attempts to unveil the mechanism underlying how place-based policies affect carbon emission. We verify that economic development, industrial structure, and technological progress are the three main mechanisms. Our results contribute significantly to the body of knowledge on place-based policies and can offer suggestions for future optimization of place-based policies and governance in other areas.

This study is organized structurally as follows. Section 2 explains the ORDP’s background and constructs the theoretical hypothesis. Section 3 provides the methodology and data. Section 4 reports the baseline outcomes with a series of robustness tests, mechanisms, and heterogeneity analysis of OPRD on carbon emission. Section 5 presents conclusions and related policy suggestions.

## 2 Policy background and theoretical framework

### 2.1 Policy background

Old Revolutionary Regions are revolutionary base areas founded under the Communist Party of China’s leadership during the Agrarian Revolutionary War and the Anti-Japanese War, which cover more than 1, 300 counties (cities and districts) in 28 provinces around mainland China, occupying one-third of China’s total land area. Compared with other regions in China, old revolutionary regions are in a relatively lagging stage of development. Therefore, in recent years, the Chinese government has given a high priority to supporting the revitalization and development of old revolutionary regions. ORDP is one of the most massive supportive programs.

**Fig. 2:**
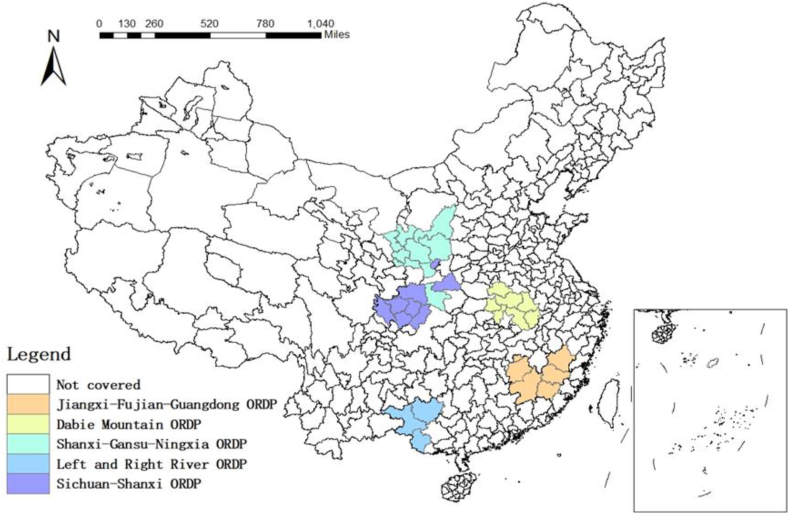
The geographical distribution of ORDP cities.

Since 2012, China has introduced strategies for the revitalization and development of five key revolutionary old areas, including Shanxi-Gansu-Ningxia, Jiangxi-Fujian-Guangdong, Dabie Mountains, Left and Right River, and Sichuan-Shanxi old Revolutionary Regions, covering 283 counties (cities and districts), with an area of 845,600 square kilometers and involving 166.7 million people (as Fig. 2 shows). When the “13th Five-Year Plan” was in effect, one out of eight of the central funds were used in these five old revolutionary areas, with an investment scale of more than RMB 300 billion.

ORDP provides a series of financial, fiscal, industrial, and talent policies in the old revolutionary regions, which may have impacts on carbon emission. For example, ORDP proposes to strengthen oil and gas exploration and increases investment in infrastructure construction in the old revolutionary regions. Exploration for oil and gas results in higher carbon emission [27]. While promoting social and economic advancement, infrastructure development can also significantly increase carbon emission [28].

### 2.2 Theoretical framework

#### 2.2.1 ORDP’s impact on carbon emission

As we mentioned in the introduction section, the impact of place-based policies on carbon emission is unclear, it can either increase or reduce carbon emission. However, based on the specific measures implemented in ORDP areas, we propose our **Hypothesis 1** that ORDP will lead to an increase in carbon emission. The reasons are as follows.

Unlike other place-based policies, such as China’s special economic zones, which always targeted regions that have a stronger economic base or favorable geographic conditions [21], ORDP specifically targets economically impoverished and geographically disadvantaged areas [12]. For these ORDP areas, the primary goal is to enhance economic development and people’s welfare. Therefore, the policies implemented in ORDP mainly focus on economic development. For example, most financial investments in the ORDP are distributed in industrial development, transportation improvement, and infrastructure construction of the ORDP areas, providing important financial guarantees for the ORDP area’s economic development [29,30]. According to the Environmental Kuznets Curve (EKC) hypothesis proposed by Grossman G.M. et al. [31], there is an inverted U-shaped relationship between economic growth and environmental pollution, that is, when a region is at the stage of economic development, the level of environmental pollution increases with economic growth; however, when economic development reaches a certain level, namely a turning point, the level of environmental pollution tends to decrease. Due to a weaker economic base, ORDP areas are at the stage of economic development, therefore, according to EKC, carbon emission in ORDP areas tends to increase with economic development. In addition, the emission reduction technologies in these ORDP areas are relatively backward compared to other areas [32], which also lead to higher carbon emission. Therefore, given advancing economic development and trailing emission reduction technology, the implementation of ORDP will cause the growth of carbon emission.

However, we also propose that the impact of ORDP on carbon emission will take a period to emerge and is not sustainable in the long term, which is our **Hypothesis 2**. The reasons for this Hypothesis are as follows. First, the policies implemented in ORDP need to be given a certain amount of time for the local government and enterprises to respond and thus it takes a period for the effect of ORDP to emerge. Second, ORDP realizes its policy effect primarily through massive infrastructure construction, physical investment, and industrial development, rather than by technological progress or human capital investment. These measures may have a short-term effect on economic development and carbon emission, but this effect may gradually reduce in the long term.

#### 2.2.2 The mechanisms of ORDP on carbon emission

In the above subsections 2.2.1, we analyze theoretically the role of ORDP in carbon emission, but what are the mechanistic pathways of ORDP increasing carbon emission? Clarifying this issue has important implications for the environment and economic development of developing countries such as China. Following the studies by Grossman G.M. et al. [31], which divide the environmental effects of economic activity into scale, structural, and technological effects, we propose our **Hypothesis 3** that the impact of ORDP on carbon emission is mainly caused by scale effect, structural effect, and technological effect. The following are detailed explanations.

First, ORDP will promote the economic development of the ORDP areas, leading to an increase in carbon emission. As we stated above, the fundamental target of ORDP is to enhance economic development. ORDP issues many policies to encourage economic development in the old revolutionary regions. Previous studies have verified that economic development will result in environmental pollution, such as carbon emission [33]. Just as Färe R. et al. [34] pointed out that “there is no fire without smoke”, in other words, there may be no economic development without environmental contamination. Moreover, compared to other areas with efficient energy utilization, ORDP areas with poor development in many aspects tend to be inefficient in energy utilization, so the causal relationship between economic development and carbon emission becomes more apparent [35]. Therefore, given the same level of emission reduction technology and the current economic development level of the ORDP areas, an increase in the scale of economic output will certainly result in a proportional rise in carbon emission.

Second, ORDP changes the industrial structure and promotes secondary industry development in ORDP areas, resulting in carbon emission growth [33]. The ORDP areas have abundant resources including coal, petroleum, and natural gas, providing opportunities for the growth of energy-intensive industries [12]. For example, Shanxi-Gansu-Ningxia is abundant in energy resources and the government encourages the development of large-scale coal majors, the construction of coal-fired power stations, and the active expansion of natural gas resources in this region. The revitalization plans for the Left and Right River, Sichuan-Shanxi, Dabie Mountains, and Jiangxi-Fujian-Guangdong explicitly indicate the importance of accelerating the upgrading of manufacturing industries, promoting tourism, and building resource refinement processing bases. Moreover, preferential tax policies have attracted local and foreign capital to invest in these energy-intensive industries [3]. It is reported that industrial added value in ORDP areas increases from RMB 57.5 trillion to RMB 120.1 trillion from 2010 to 2019 [7]. Under the condition that the energy utilization technology is generally backward in ORDP areas, the restructuring of the industrial structure with an increasing share of the secondary industry will lead to a growth in carbon emission [36].

**Fig. 3:**
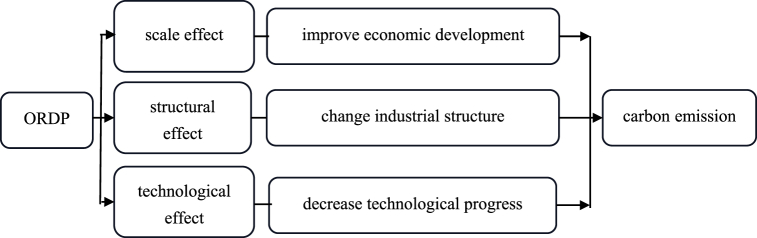
The mechanisms of ORDP on carbon emission.

Third, previous studies have shown that technological progress has a favorable impact on environmental quality, which means a decrease in carbon emission [37]. However, the implementation of ORDP fails to significantly improve technological progress [32]. Because the policies in ORDP tend to focus on physical capital investment and infrastructure development the effects of these investments are more obvious to be observed. In contrast, investments in education, human capital, and science and technology, which can facilitate technological progress, tend to require a long-term and substantial investment to have a significant impact. So government investment in these areas tends to be inadequate when implementing ORDP. All of the above can result in inefficient technological innovation in the ORDP areas, thereby inhibiting the impact of technological progress on carbon emission reduction. Fig. 3 expresses three mechanisms of ORDP on carbon emission.

## 3 Research design

### 3.1 Methodology

This study aims to assess the causal relationship between ORDP and carbon emission. The multiple regression method is often used to catch such effects. However, this method cannot eliminate endogeneity problems, such as missing variables and time trend disturbances. A quasi-experiment based on a DID estimator can well address these endogeneity problems; hence the causal effect can be recognized accurately. A traditional DID method requires that the policy must begin to implement at the same time, but ORDP in this study was introduced and implemented in batches over years 2012–2016, which means that the policies impacted ORDP areas at different times, Therefore, we use the time-varying DID approach to catch the effect of ORDP on carbon emission. Following Beck T. et al. [38], we adopt the following model (1) to examine the average treatment effect of ORDP on carbon emission:

(1) $${CE}_{it}=\alpha_{0}+\alpha_{1}{ORDP}_{it}+\rho X_{it}+\mu_{i}+\delta_{t}+\varepsilon_{it}$$

where, ${CE}_{it}$ reflects i city’s carbon emission in year t. ${ORDP}_{it}$ indicates the treatment variable, which is 1 if city i at year t implemented ORDP and 0 otherwise. $X_{it}$ denotes a range of control variables that affect carbon emission. $\mu_{i}$ and $\delta_{t}$ denote city fixed effect and time fixed effect, and $\varepsilon_{it}$ is the random error term. If $\alpha_{1}$ is significantly positive, indicating that our hypothesis holds that ORDP leads to an increase in carbon emission.

Model (1) can only measure the average treatment effect of ORDP on carbon emission from a static standpoint, it does not account for the dynamic change of effects over time. Therefore, we adopt an event analysis method to evaluate the dynamic treatment effect of ORDP on carbon emission. The model (2) is as follows:

(2) $${CE}_{it}=\alpha_{0}+\sum_{k\geq -6}^{5}\alpha_{k}{ORDP}_{it}^{k}+\rho X_{it}+\mu_{i}+\delta_{t}+\varepsilon_{it}$$

where ${ORDP}_{it}^{k}$ is a sequence of “event-time” dummy variables indicating k years from the ORDP implementation. The dummy variable ${ORDP}_{it}^{k}=1$ represents the post-ORDP city, otherwise ${ORDP}_{it}^{k}=0$. $\alpha_{k}$ is the coefficient of ${ORDP}_{it}^{k}$, representing the effect of ORDP on carbon emission after ORDP implementation in year k. The other variables are identical to model (1).

### 3.2 Variable measurements

#### 3.2.1 Measurement of carbon emission

We use industrial carbon emission to represent carbon emission in China since the industrial sector contributes to more than 70% of the total emissions in China. Therefore, according to the method proposed by Han F. et al. [39], we employ the following model (3) to calculate industrial carbon emission:

(3) $$CE=C_{n}+C_{p}+C_{e}=kE_{n}+\gamma E_{p}+\varphi \left(\delta \times E_{e}\right)$$

where CE is the total industrial carbon emission, $C_{n}$, $C_{p}$, and $C_{e}$ stand for industrial carbon emission from the consumption of natural gas, liquefied petroleum gas (LPG), and electricity, respectively. $E_{n}$, $E_{P}$, and $E_{e}$ denote the industrial consumption of natural gas, LPG, and electricity, respectively. k, γ, and φ express the carbon emission coefficient of natural gas, LPG, and coal power fuel chain, which are equal to 2.162 kg/m^2^, 3.101 kg/kg, and 1.302 kg/(kW·h), respectively. δ is the share of coal-powered electricity. According to China Electricity Yearbook 2010 to 2019, the value of δ from 2010 to 2019 is 80.8%, 82.4%, 78.7%, 78.6%, 75.8%, 73.7%, 71.8%, 71.1%, 70.4%, and 69%, respectively.

According to Sun P. et al. [40], we apply the logarithms of industrial total carbon emission (ToTCO_2_) and carbon emission per 10,000 yuan of industrial value-added (PerCO_2_) to measure carbon emission.

#### 3.2.2 Independent variables

We treat ORDP as the core explanatory variable. We define ORDP for a city in year t and following years as 1 if the ORDP is applied in the city, and 0 otherwise.

#### 3.2.3 Control variables

To avoid the endogeneity problem that affects the research results due to omitted variables, we control some factors that affect carbon emission. Based on the existing studies, we choose the subsequent four indicators as control variables: economic development, urbanization level, private saving, and human capital. The specifics are as follows.

(1) Economic development. We measured it using the logarithm of real GDP. Economic development is an important factor influencing carbon emission. On the one hand, economic development contributes to massive energy consumption and thus increases carbon emission [41]. On the other hand, economic development can encourage the exploitation and utilization of clean energy as it increases people’s concerns about environmental conservation, which contributes to carbon emission reduction [42].
(2) Urbanization level. Determined by dividing the total population by the fraction of urban residents. Academics are controversial concerning the effect of urbanization on carbon emission. Some consider that urbanization will increase carbon emission in that it will raise people’s income and enhance their consumption capacity [43]. Others believe that urbanization contributes to carbon reduction in that it will promote an upgrade of people’s consumption direction towards service products with low carbon emission [44].
(3) Private saving. We express it in the logarithm of year-end resident deposits. The impact of private saving on carbon emission is controversial. On the one hand, private saving boosts carbon emission. Because when society contains more private savings, the investment in the economy is also higher, this is beneficial to economic growth. With the rapid economic development, carbon emission will increase [45]. On the other hand, private saving may also lead to carbon emission reduction. Because increasing private saving means less consumption and a smaller market scale, which is detrimental to economic development [46].
(4) Human capital. Defined by a logarithmic based on the total number of college students. Uncertainty exists on how human capital affects carbon reduction. On the one hand, an improvement in human capital level results in carbon emission reduction, because it upgrades industrial structure, and promotes economic growth [47]. On the other hand, a higher human capital level increases carbon emission. Because human capital leads to industrial clustering and population concentrations, which increase energy consumption demand and further result in carbon emission [48].

Table 1 displays the definitions and statistics for the aforementioned variables. Total original variable data in this study are obtained from the 2010–2019 “China City Statistical Yearbook”, “China Regional Economic Statistical Yearbook”, and “China Electricity Yearbook”. For some missing data, we manually compile provincial and local statistical yearbooks as well as statistical bulletins to find gaps. The remaining missing data are filled by linear interpolation.

**Table 1:** Variable descriptive statistics.

Variable	Definition	Mean	S.D.	N
ToTCO_2_	The logarithm of total industrial carbon emission	21.894	1.236	1099
PerCO_2_	The logarithm of carbon emission per 10,000 yuan of industrial value-added	1.823	1.067	1099
ORDP	=1, if city i at year t implemented ORDP; =0, otherwise	0.184	0.387	1100
Economic development	The logarithm of real GDP	16.042	0.823	1100
Urbanization level	The share of urban residents in the overall population	0.219	0.271	1100
private saving	The logarithm of year-end resident deposits	15.847	0.952	1100
Human capital	The logarithmic based on the total number of college students	7.229	14.621	1100

### 3.3 Sample selection

Based on the comprehensive consideration of the rational selection of research samples and the available data, this paper selects 110 prefecture-level cities in the scope of ORDP areas as our research samples. Specifically, 33 prefecture-level cities—15 in 2012, 10 in 2015, and 8 in 2016—are treated as the experimental group, and the rest 77 prefecture-level cities are recognized as control groups. Due to data availability, our research period is from 2010 to 2019.

## 4 Empirical results

In this part, firstly, we assess the average treatment effect of ORDP on carbon emission employing the DID approach, and then investigate the dynamic treatment effect of ORDP on carbon emission based on the event study method; in addition, we conduct some robustness tests; and then we test the underlying mechanisms of ORDP on carbon emission. Finally, we examine the heterogeneous impact of ORDP on carbon emission across cities in different regions.

### 4.1 ORDP’s average treatment effect on carbon emission

Based on the model (1), we estimate the average treatment effect of ORDP on carbon emission, and the baseline estimation outcomes are displayed in Table 2. The estimated outcomes are displayed in columns (1) and (3) with no control variables. The coefficient of ORDP in column (1) is 0.284 and significant at the 1% level, which represents that ORDP leads to an increase in ToTCO_2_. The ORDP coefficient in column (3) is 0.223 and significant at the 5% level, demonstrating that ORDP raises PerCO_2_. Furthermore, to acquire a more robust result, we control some factors that influence carbon emission. The estimated results including the control variables are shown in columns (2) and (4). The results indicate that the increasing effect of ORDP on carbon emission persists, with an increase in ToTCO_2_ by 26.7% at the 1% significance level and an increase in PerCO_2_ by 18.4% at the 5% significance level. Therefore, we conclude that ORDP significantly increases carbon emission, which validates our Hypothesis 1.

**Table 2:** The average treatment effect of ORDP on carbon emission.

	ToTCO_2_		PerCO_2_	
	(1)	(2)	(3)	(4)
*ORDP*	0.284***	0.267***	0.223**	0.184**
	(0.092)	(0.093)	(0.094)	(0.094)
*Urbanization level*		0.048		−0.012
		(0.042)		(0.050)
*Private saving*		0.039*		0.013
		(0.021)		(0.029)
*Human capital*		−0.011		−0.012
		(0.007)		(0.008)
*Economic development*		−0.120		−0.613***
		(0.140)		(0.158)
*City Fixed effects*	Yes	Yes	Yes	Yes
*Year Fixed effects*	Yes	Yes	Yes	Yes
*R*^*2*^	0.768	0.768	0.662	0.671
*Observations*	1099	1099	1099	1099

*Notes:* Robust standard errors clustered at the city level are reported in parentheses; ***, **, and * indicate statistical significance at 1%, 5%, and 10%, respectively, same as below.

### 4.2 The dynamic treatment effect of ORDP on carbon emission

The baseline results estimate ORDP’s average treatment effect on carbon emission based on a static perspective only. However, considering that controlling carbon emission is a necessary part of sustainable economic development in the long term, ORDP’s dynamic treatment effect on carbon emission also merits attention.

Based on the model (2), we examine the dynamic treatment effect of ORDP on carbon emission, and Table 3 displays the results. We discover that the impact of ORDP on carbon emission takes a period to emerge and is not sustainable in the long term, which validates our Hypothesis 2. Specifically, the coefficients of ${ORDP}_{0}$, ${ORDP}_{1}$, and ${ORDP}_{2}$ are not significant, demonstrating that the impact of ORDP on carbon emission, is insignificant in the current, first, and second years of ORDP deployment. The coefficients of ${ORDP}_{3}$, and ${ORDP}_{4}$ are significant and show a significant upward trend, suggesting that the impact of ORDP on carbon emission reaches the maximum value in the fourth year of ORDP implementation. However, the coefficient of ${ORDP}_{5}$ is not significant, revealing that the impact of ORDP on carbon emission is not long-lasting.

**Table 3:** The dynamic treatment effects of ORDP on carbon emission.

	ToTCO_2_		PerCO_2_	
	(1)	(2)	(3)	(4)
*ORDP*_*-6*_	0.086	0.108	0.296	0.277
	(0.220)	(0.222)	(0.199)	(0.197)
*ORDP*_*-5*_	0.025	0.043	0.104	0.130
	(0.163)	(0.162)	(0.160)	(0.155)
*ORDP*_*-4*_	−0.089	−0.080	−0.018	0.004
	(0.185)	(0.185)	(0.190)	(0.187)
*ORDP*_*-3*_	0.149	0.155	0.207	0.224
	(0.213)	(0.213)	(0.213)	(0.212)
*ORDP*_*-2*_	−0.165	−0.164	−0.119	−0.126
	(0.184)	(0.184)	(0.189)	(0.187)
*ORDP*_*0*_	0.238	0.236	0.233	0.236
	(0.157)	(0.158)	(0.159)	(0.157)
*ORDP*_*1*_	0.031	0.023	0.011	−0.001
	(0.159)	(0.157)	(0.157)	(0.150)
*ORDP*_*2*_	0.245*	0.212	0.190	0.077
	(0.135)	(0.136)	(0.131)	(0.133)
*ORDP*_*3*_	0.364***	0.357**	0.331**	0.326**
	(0.140)	(0.140)	(0.140)	(0.138)
*ORDP*_*4*_	0.439***	0.431***	0.416**	0.407**
	(0.150)	(0.150)	(0.162)	(0.158)
*ORDP*_*5*_	0.137	0.069	0.500	0.279
	(0.277)	(0.271)	(0.350)	(0.337)
*City Fixed effects*	Yes	Yes	Yes	Yes
*Year Fixed effects*	Yes	Yes	Yes	Yes
*Control variables*	No	Yes	No	Yes
*R*^*2*^	0.769	0.769	0.664	0.674
*Observations*	1099	1099	1099	1099

*Note:* The same as Table 2.

### 4.3 Robustness tests

To improve the reliability of our results, we conduct seven robustness tests, containing a common trend test, a placebo test, a randomness test, advancing the time of ORDP implementation, changing the definition of ORDP, employing the propensity score matching and the difference-in-differences (PSM-DID) model, and eliminating the interference of other policies.

#### 4.3.1 Test of common trend

The presence of a common trend in carbon emission between ORDP cities and non-ORDP cities before ORDP implementation is an essential prerequisite for estimating the net effect of the ORDP on carbon emission using the DID approach. Therefore, it is necessary to examine the common trends or pre-existing trends.

**Fig. 4:**
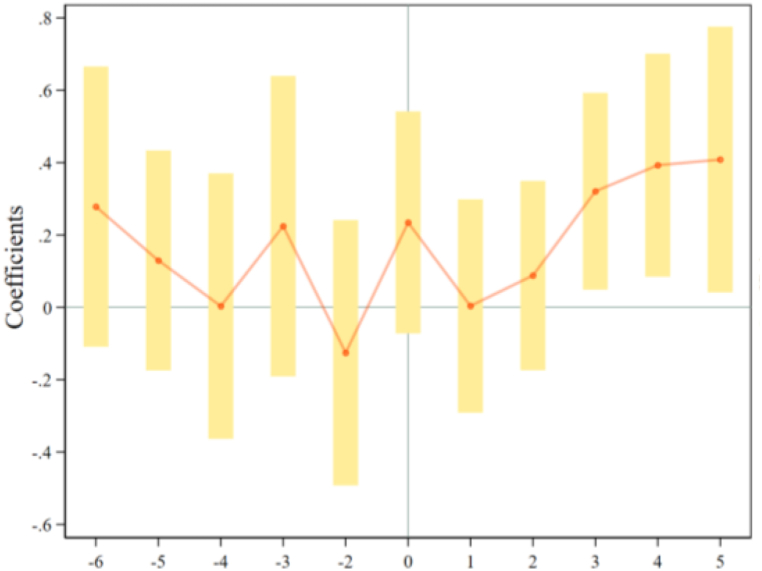
The common trend test of ToTCO_2_.

**Fig. 5:**
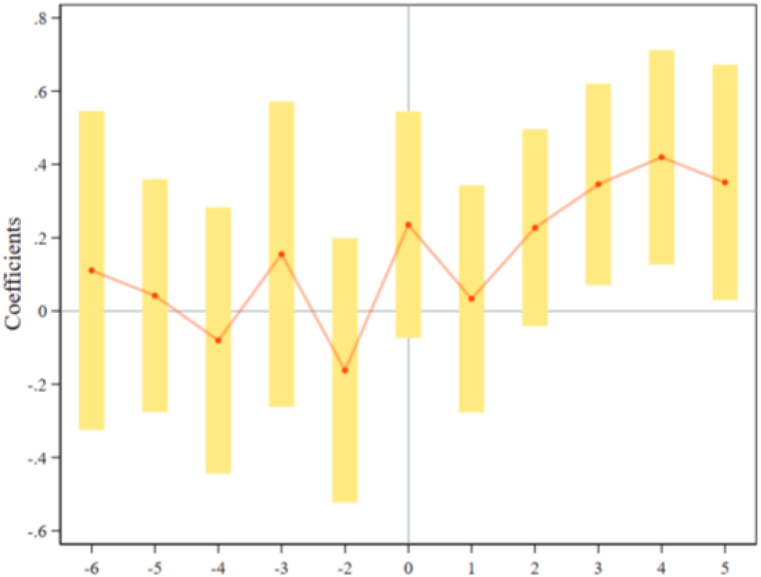
The common trend test of PerCO_2_.

Fig. 4, Fig. 5 express the pre-existing trend test results of ToTCO_2_ and PerCO_2_, respectively. We find that the coefficients of ORDP for all years are non-significant at the 90% confidence interval before the ORDP implementation, which means the common trend exists and our research design passes the pre-existing trend test. Therefore, we can explore how ORDP influences carbon emission using the DID model.

#### 4.3.2 Placebo test

To remove any possible effects of observable factors on the baseline results, we perform a placebo test according to Li P. et al. [49]. Specifically, we construct a false ORDP variable by randomly selecting the year and the cities of ORDP implementation. Then we repeat the regression based on model (1) by using the false-ORDP variable. We perform this process for 500 replications.

**Fig. 6:**
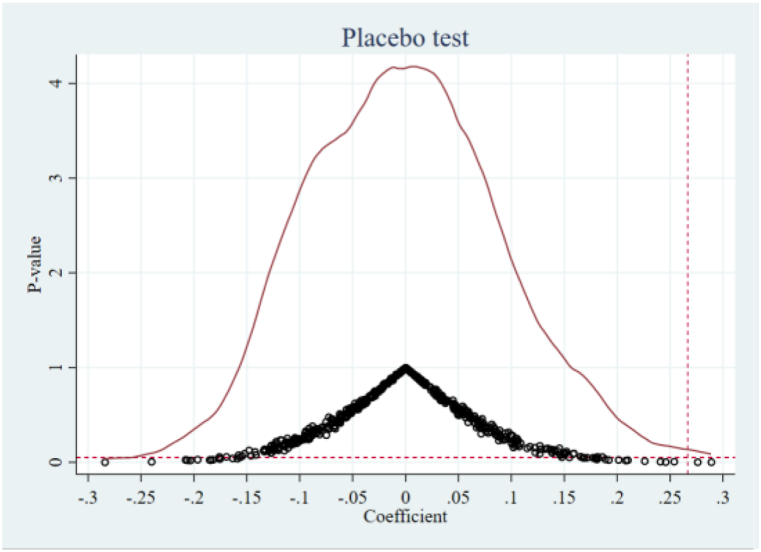
Placebo testing of ToTCO_2_.

**Fig. 7:**
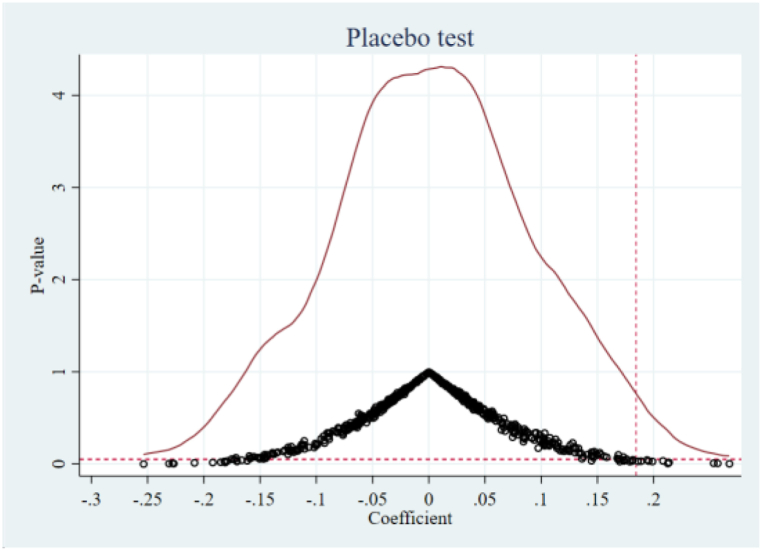
Placebo testing of PerCO_2_.

The probability density distribution of the placebo ORDP variable’s coefficients for ToTCO_2_ and PerCO_2_ are displayed in Fig. 6, Fig. 7, respectively. We discover that the placebo ORDP variable’s coefficients are distributed centrally around zero, while our baseline regression findings in Table 2 (marked by the red vertical dashed lines representing the values of 0.267 and 0.184) are significantly away from this center. The placebo test results demonstrate that ORDP’s impact on carbon emission is unlikely to be affected by other unobservable factors, which strengthens our confidence in our findings.

#### 4.3.3 Randomness test

The deployment of ORDP is assumed to be unaffected by initial carbon emissions in different cities in our empirical research. However, local governments may choose when and where to implement ORDP based on each city’s carbon emission status. Thus, the implementation of ORDP may have a selection bias problem. We conduct a randomness test to prove that the cities in ORDP are not chosen based on their initial carbon emission. Referring to Shen K.R. et al. [50], to evaluate the randomness of ORDP, we employ the following model (4):

(4) $$policy\_year=\tau {CE}_{i}^{2010}+\rho X_{i}^{2010}+\varepsilon_{i}$$

where policy_year is the year when city i implements ORDP, ${CE}_{i}^{2010}$ is the city’s carbon emission in 2010 (the initial stage of our sample), $X_{i}^{2010}$ refers to the value of the control variables in 2010, and $\varepsilon_{i}$ represents random error. According to the regression findings of the randomness test presented in Table 4, there is no discernible connection between carbon emissions and the year ORDP was introduced, which further indicates that the implementation of ORDP is random and the baseline regression shows no glaring selection bias.

**Table 4:** Randomness test results of ORDP on carbon emission.

	policy_year			
	(1)	(2)	(3)	(4)
*ToTCO*_*2*_	0.050	0.037		
	(0.043)	(0.100)		
*PerCO*_*2*_			0.359	0.033
			(0.223)	(0.097)
*Control variables*	No	Yes	No	Yes
*City Fixed effects*	Yes	Yes	Yes	Yes
*R*^*2*^	0.608	0.667	0.657	0.667

*Note:* The same as Table 2.

#### 4.3.4 Advancing the implementation time of ORDP

To exclude some factors before the ORDP implementation to confound our results, we also conduct a counterfactual test by constructing the false ORDP variables. Specifically, first, we advance the implementation time of ORDP by 5, 4, and 3 years, which are represented by ${ORDP}_{f5}$, ${ORDP}_{f4}$, and ${ORDP}_{f3}$, respectively. Then, we use these false ORDP variables to regress the model (1). If the coefficients of these false ORDP variables are insignificant, it means that the implementation of ORDP indeed causes an increase in carbon emission; otherwise, it is produced by unobservable elements, resulting in a non-robust conclusion. The regression results from Table 5 demonstrate that the coefficients of ToTCO_2_ and PerCO_2_ are not significant with or without control variables, indicating that the increase in carbon emission is indeed attributable to ORDP and our baseline regression results are robust.

**Table 5:** The regression results of advancing ORDP implementation time.

	ToTCO_2_		PerCO_2_	
	(1)	(2)	(3)	(4)
*ORDP*_*f3*_	0.047	0.031	−0.053	−0.082
	(0.101)	(0.101)	(0.084)	(0.083)
*ORDP*_*f4*_	0.101	0.084	0.002	−0.022
	(0.130)	(0.130)	(0.135)	(0.130)
*ORDP*_*f5*_	−0.004	−0.024	−0.109	−0.127
	(0.141)	(0.140)	(0.135)	(0.130)
*Control variables*	No	Yes	No	Yes
*City Fixed effects*	Yes	Yes	Yes	Yes
*Year Fixed effects*	Yes	Yes	Yes	Yes
*Observations*	1099	1099	1099	1099
*R*^*2*^	0.765	0.766	0.659	0.670

*Note:* The same as Table 2.

#### 4.3.5 Changing the definition of ORDP

Considering the differences in the specific implementation month of ORDP in the five old revolutions, this paper refers to Lu Y. et al. [51] to change the definition of ORDP to conduct a robustness test. Specifically, for ORDP cities in Shanxi-Gansu-Ningxia old revolution with an implementation date of March 25, 2012, we assign ${ORDP}_{it}$ to 0 before 2012 and 3/4 in 2012. For ORDP cities in the Jiangxi-Fujian-Guangdong old revolution with the implementation time of June 28, 2012, ${ORDP}_{it}$ is assigned to a value of 0 if the year is before 2012, and a value of 1/2 in 2012. For ORDP cities in the Left and Right River old revolution with the specific implementation date of February 9, 2015, the value of the ${ORDP}_{it}$ is 0 before 2015, and 11/12 in 2015. For the ORDP cities in the Dabie Mountain old revolution with the implementation time of June 5, 2015, the value of the ${ORDP}_{it}$ is 0 before 2015 and 7/12 in 2015. For the ORDP cities in the Sichuan-Shanxi old revolution with the implementation time of August 3, 2016, the value of the ${ORDP}_{it}$ is 0 before 2016 and 5/12 in 2016. The estimate outcomes are presented in Table 6, and we discover that our baseline results are robust since the coefficients of ${ORDP}_{it}^{\prime }$ are insignificantly different from the estimated coefficients of the baseline regression.

**Table 6:** Changing the definition of ORDP with consideration of the difference in policy implementation months.

	ToTCO_2_		PerCO_2_	
	(1)	(2)	(3)	(4)
${ORDP}_{it}^{\prime }$	0.280***	0.260***	0.219**	0.173*
	(0.097)	(0.098)	(0.099)	(0.099)
*City Fixed effects*	Yes	Yes	Yes	Yes
*Year Fixed effects*	Yes	Yes	Yes	Yes
*Control variables*	No	Yes	No	Yes
*Observations*	1099	1099	1099	1099
*R*^*2*^	0.768	0.768	0.661	0.671

*Note:* The same as Table 2.

#### 4.3.6 PSM-DID method

The baseline results directly use DID method to analyze the distinctions in carbon emission between ORDP and non-ORDP cities. However, systematic differences may exist between ORDP and non-ORDP cities before the implementation of ORDP. Referring to Zhang C. et al. [7], a combination of PSM and DID is used for robust estimation. The PSM-DID method first matches cities that implemented ORDP similarly to cities not implemented ORDP by selecting appropriate matching variables. Then we estimate ORDP’s effects based on the DID method.

Specifically, the first step of PSM-DID is to compute the propensity score for each observation sample based on the logit regression coefficients, where the ORDP dummy is the explanatory variable, and four control variables shown in Table 1 are taken as covariates. The second step is matching non-ORDP cities based on annual panel data using radius matching, kernel matching, and nearest-neighbor matching methods using the propensity scores obtained in the first step [52]. Eventually, the impacts of ORDP on carbon emission are re-identified based on the matched sample using model (1).

The outcomes of the PSM-DID method are shown in Table 7. All outcomes demonstrated that PSM-DID estimations match the baseline regression results identically, further demonstrate the robustness of our baseline results, and suggest that ORDP indeed significantly increases carbon emission.

**Table 7:** Results of the PSM-DID method.

	Radius matching	Kernel matching	Nearest-neighbor matching
***ToTCO***_***2***_			
*ORDP*	0.278***(0.100)	0.278***(0.096)	0.252**(0.099)
*R*^*2*^	0.783	0.772	0.776
*Observations*	747	986	768
***PerCO***_***2***_			
*ORDP*	0.221**(0.100)	0.216**(0.096)	0.187*(0.100)
*R*^*2*^	0.723	0.707	0.715
*Observations*	747	986	768
*Control variables*	Yes	Yes	Yes
*City Fixed effects*	Yes	Yes	Yes
*Year Fixed effects*	Yes	Yes	Yes

*Note:* The same as Table 2.

#### 4.3.7 Eliminating the interference of other policies

Other policies that can also influence carbon emission might skew the impact of ORDP. Therefore, for the baseline regression results to be credible and robust, we must eliminate the interference of other policies. By combing existing literature and policies, we identify two policies—the Targeted Poverty Alleviation Policy (TPA) and the National Innovation City Pilot Policy (NICP), which occurred during our research period—that may influence carbon emission and thus distort our evaluation results.

Firstly, TPA can promote economic development and may affect carbon emission. To achieve poverty eradication among the rural poor, China has implemented TPA since 2013. Significant funding is distributed to industrial development, transportation improvement, and infrastructure construction in TPA counties, which may affect carbon emission.

Secondly, NICP, aiming to foster innovative cities, will also have an impact on carbon emission. To increase innovation abilities and reach sustainable social development, China has implemented NICP since 2008. Up to 2020, 78 cities have implemented NICP [53]. Through NICP, technological innovation can be enhanced and thus can upgrade energy conservation and emission reduction technologies, which may affect carbon emission.

Following Li P. et al. [49], we incorporate the dummy variables of these two policies into our model and re-estimate the effect of ORDP on carbon emission based on model (1) after eliminating the interference of these two policies. If the ORDP’s coefficient remains positive and is consistent with the baseline results, it demonstrates that these two policies have no impact on the conclusion of our paper.

The regression results, which are shown in Table 8, are almost identical to our baseline results. It demonstrates that ORDP increases carbon emission even after the effects of the two aforementioned policies are eliminated, further demonstrating the validity of our empirical findings.

**Table 8:** Eliminating the interference of other policies.

	Eliminate the interference of TPA	Eliminate the interference of NICP
***ToTCO***_***2***_		
*ORDP*	0.339***(0.100)	0.281***(0.093)
*TPA*	−0.299**(0.137)	
*NICP*		0.075 (0.070)
*R*^*2*^	0.770	0.769
*Observations*	1098	1098
***PerCO***_***2***_		
*ORDP*	0.257**(0.099)	0.189**(0.094)
*TPA*	−0.342**(0.138)	
*NICP*		0.035 (0.071)
*R*^*2*^	0.675	0.672
*Observations*	1098	1098
*Control variables*	Yes	Yes
*City Fixed effects*	Yes	Yes
*Year Fixed effects*	Yes	Yes

*Note:* The same as Table 2.

### 4.4 Mechanism analysis

The investigation mentioned above has established that ORDP causes an increase in carbon emission. The following question is what mechanisms lead to this result? In other words, what are the transmission mechanisms of ORDP on carbon emission? As we stated in section 2.2 the influence of ORDP on carbon emission is mainly due to the scale effect, structural effect, and technological effect. Therefore, we adopt a three-step methodology from Tofighi D. et al. [54] to empirically investigate the aforementioned three mechanisms. These are the particular models (5)–(7):

(5) $${CE}_{it}=\alpha_{0}+\alpha_{1}{ORDP}_{it}+\rho X_{it}+\mu_{i}+\delta_{t}+\varepsilon_{it}$$

(6) $$M_{it}=\beta_{0}+\beta_{1}{ORDP}_{it}+\varphi_{1}X_{it}+\mu_{i}+\delta_{t}+\varepsilon_{it}$$

(7) $${CE}_{it}=\theta_{0}+\vartheta_{1}{ORDP}_{it}+\vartheta_{2}M_{it}+\varphi_{2}X_{it}+\mu_{i}+\delta_{t}+\varepsilon_{it}$$

where $M_{it}$ represents the three mechanisms, which are scale effect, structural effect, and technological effect. $\alpha_{1}$ denotes ORDP’s total effect on carbon emission; $\vartheta_{1}$ indicates ORDP’s direct effect on carbon emission; $\beta_{1}\times \vartheta_{2}$ is ORDP’s indirect effect on carbon emission. Referring to Friedl B. et al. [55], the scale effect refers to the growth in production scale accompanying economic growth, which can be expressed in terms of per capita GDP, the structural effect means an increase in the proportion of industry in the economic structure, which can be denoted as the share of secondary industrial output in regional GDP, and the technological effect represents the pursuit of new techniques to enhance the competitiveness and profitability of companies to improve energy utilization efficiency, which can be measured by Total Factor Productivity (TFP). According to Färe R. et al. [56], we use the Malmquist index method to measure TFP. The following is the specific model (8):

(8) $$M_{i}^{t}\left(x^{t+1},y^{t+1};x^{t},y^{t}\right)={\left\{\left[\frac{D_{i}^{t}\left(x^{t},y^{t}\right)}{D_{i}^{t}\left(x^{t+1},y^{t+1}\right)}\right]\left[\frac{D_{i}^{t+1}\left(x^{t},y^{t}\right)}{D_{i}^{t+1}\left(x^{t+1},y^{t+1}\right)}\right]\right\}}^{1/2}$$

where $M_{i}^{t}$ refers to TFP from period t to t+1, $\frac{D_{i}^{t}\left(x^{t},y^{t}\right)}{D_{i}^{t}\left(x^{t+1},y^{t+1}\right)}$ is the change in technical efficiency from period t to t+1 at the level of technology in period t, $\frac{D_{i}^{t+1}\left(x^{t},y^{t}\right)}{D_{i}^{t+1}\left(x^{t+1},y^{t+1}\right)}$ reflects the change in technical efficiency from period t to t+1 at the level of technology in period t+1. We use GDP as output, labor and capital stock as inputs, to measure TFP. Labor is measured by the employee numbers in each city at the year-end. Capital stock is measured by using the perpetual inventory method and is expressed as follows model (9):

(9) $$K_{t}=I_{i,t}+\left(1-\delta \right)K_{t-1}$$

where $K_{t}$ is the capital stock in year t, $I_{i,t}$ represents the total amount of additional fixed capital added to the society in year t, δ indicates the depreciation rate, setting as 10% as Pan D. et al. [57] did. These three variables are all measured using natural logarithms.

The following are the specific test procedures. First, we identify the significance of $\beta_{1}$ and $\vartheta_{2}$. The mediating effect is demonstrated if both coefficients are significant and $\beta_{1}\times \vartheta_{2}$ has the same sign as $\vartheta_{1}$. Second, we apply the Bootstrap test to check whether $\beta_{1}\times \vartheta_{2}=0$ if at least one of $\beta_{1}$ and $\vartheta_{2}$ is not significant. If the outcome is significant and $\beta_{1}\times \vartheta_{2}$ has the same sign as $\vartheta_{1}$, the mediating impact is likewise proven.

Table 9 presents the outcomes. The total impact of ORDP on carbon emissions is displayed in column (1). We examine each of the three mechanisms independently from columns (2) to (7). First, the outcomes of economic development are displayed in columns (2)–(3). The ORDP has accelerated economic development, as $\beta_{1}$ (0.070) is significant at the 1% level. $\vartheta_{2}$ (0.530) is also significant. $\beta_{1}\times \vartheta_{2}$ has a positive sign, which is consistent with $\vartheta_{1}$ (0.229). The above results show that economic development mediates the effect of ORDP on carbon emission. Secondly, the results of the industrial structure are shown in columns (4)–(5). $\beta_{1}$ and $\vartheta_{2}$ are both significant and $\beta_{1}\times \vartheta_{2}$ has the same sign as $\vartheta_{1}$, indicating that a mediating role is also played by industrial structure. Finally, the results of the effect of technological progress are shown in columns (6)–(7). As we can see, $\beta_{1}$ and $\vartheta_{2}$ are both significant and $\beta_{1}\times \vartheta_{2}$ has the same sign as $\vartheta_{1}$, indicating that technological progress is also a mechanism.

**Table 9:** The mechanism analysis of ORDP on carbon emission.

		Scale effect		Structural effect		Technology effect	
	(1)	(2)	(3)	(4)	(5)	(6)	(7)
	ToTCO_2_	Economic development	ToTCO_2_	Industrial structure	ToTCO_2_	technological progress	ToTCO_2_
${ORDP}_{it}$	0.267***	0.070***	0.229**	2.993***	0.198**	−0.007**	0.261***
	(0.093)	(0.012)	(0.093)	(0.591)	(0.092)	(0.003)	(0.091)
*Economic development*			0.530**				
			(0.217)				
*Industrial structure*					0.023***		
					(0.005)		
*technological progress*							−0.542**
							(0.241)
*City Fixed effects*	Yes	Yes	Yes	Yes	Yes	Yes	Yes
*Year Fixed effects*	Yes	Yes	Yes	Yes	Yes	Yes	Yes
*Control variables*	Yes	Yes	Yes	Yes	Yes	Yes	Yes
*R*^*2*^	0.768	0.968	0.770	0.856	0.774	0.176	0.768
*Observations*	1099	1099	1099	1099	1099	1099	1099

*Note:* The same as Table 2.

### 4.5 Heterogeneity analysis

The ORDP covers five old revolutionary areas, each with different resource endowments and policy implementation efficiency, so the impact of ORDP on carbon emission may differ amongst old revolutionary regions. Therefore, we further test the regional heterogeneity effects of ORDP. Analyzing the heterogeneity effects of ORDP in different old revolutions is helpful to distinguish between old revolutions that facilitate the growth of carbon emission and others that do not, which can inform policy customization for different old revolutions.

**Fig. 8:**
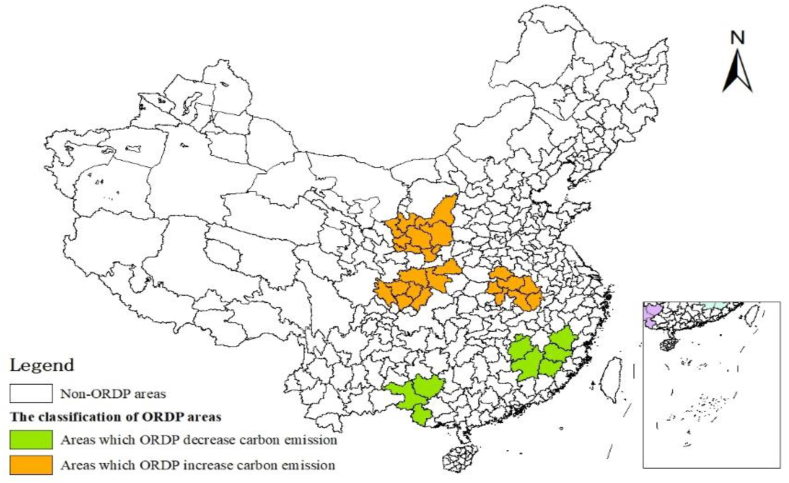
Heterogeneity analysis map of ORDP.

The influence of ORDP on carbon emission is significantly regionally heterogeneous, as seen in Table 10 and Fig. 8. Specifically, the coefficients of ORDP in Dabie Mountain, Sichuan-Shanxi, and Shanxi-Gansu-Ningxia old revolutionary regions are significantly positive, indicating that ORDP increases carbon emission in these three old revolutionary regions located in western China, which agree with the baseline results. However, the coefficients of ORDP in Jiangxi-Fujian-Guangdong, and Left and Right River old revolutionary regions are not significant, revealing that ORDP has no impact on carbon emission in these two old revolutionary regions located in central and eastern China. The possible reason for this result is that Jiangxi-Fujian-Guangdong old revolutionary region has abundant environmental resources, such as forests and water [58]. Therefore, in implementing ORDP, local governments often give priority to the development of environmental-friendly industries in this region, which is beneficial to carbon emission reduction [53]. Similarly, low-carbon industries such as the tourism industry are selected by the Left and Right River old revolutionary region as the development priority because of the advantage of its tourism resources.

**Table 10:** Results of regional heterogeneity.

	Dabie Mountain	Sichuan-Shanxi	Jiangxi-Fujian and Guangdong	Shanxi-Gansu and Ningxia	Left and Right River
*ORDP*_*it*_	0.326**	0.341**	−0.302	0.771**	−0.091
	(0.129)	(0.152)	(0.248)	(0.365)	(0.321)
*City Fixed effects*	Yes	Yes	Yes	Yes	Yes
*Year Fixed Effects*	Yes	Yes	Yes	Yes	Yes
*Control variables*	Yes	Yes	Yes	Yes	Yes
*Observations*	420	229	130	190	130
*R*^*2*^	0.779	0.817	0.752	0.727	0.711

*Note:* The same as Table 2.

## 5 Conclusion

Controlling massive carbon emission has become a topic attracting global attention and the target of all governments' endeavors. What is the place-based policy’s role in carbon emission? The answer to this question is crucial to establishing a better understanding of place-based policy’s role in carbon emission in developing countries. In this article, we use 110 prefecture-level cities from 2010 to 2019 to assess the effect of ORDP on carbon emission by considering ORDP as a natural experiment. Our results indicate that: first, ORDP can significantly increase carbon emission, and this effect takes a period to emerge and is not sustainable in the long term. Seven robustness tests demonstrate the reliability of our findings. Second, according to a mechanism analysis, the effect of ORDP on carbon emission is mainly caused by the scale effect, structural effect, and technological effect. Third, heterogeneity analysis suggests that ORDP results in a greater increased impact on carbon emission in old revolutionary cities that are located in western China compared to those located in central and eastern China.

Therefore, our paper offers the following policy implications. First, the Chinese central government should put greater emphasis on carbon emission decline and environmental management when designing specific measures in ORDP, not just only focus on promoting economic development in the revolutionary old regions. According to our baseline results, ORDP leads to an increase in carbon emission, which is harmful to the environment. Therefore, the local government in the old revolutionary regions should include environmental protection in their development objectives and pay more attention to carbon emission reduction, thus realizing a “win-win” scenario between carbon emission reduction and economic growth. Measures include establishing laws and regulations about environmental protection, increasing the supervision of polluting enterprises, raising the proportion of resource and ecological indicators in the political performance assessment, improving energy use efficiency, and so on. Second, improve technological innovation in old revolutionary areas. Our mechanism analysis shows that ORDP fails to improve technological progress and decreases the ratio of expenditure on science and technology to total government expenditure, which leads to a significant increase in carbon emission. Therefore, the government in the old revolutionary regions needs strong incentive mechanisms for technological innovation. Measures may include strengthening the investment in science and technology, accelerating the accumulation of human capital, improving the ability of ORDP to absorb and digest advanced technology and knowledge, and so on. Third, promote the industrial structure transformation of old revolutionary areas in the western region. According to the result of the heterogeneity analysis, ORDP leads to a greater increase in carbon emission in old revolutionary cities that are located in western China compared to those located in central and eastern China. Consequently, the Western regions should transform their industrial structure to create sustainable and clean industries instead of continuing to promote the expansion of energy-intensive industries.

With much work still to be done. Although the results of our paper, which examines the impacts of ORDP on carbon emission over a relatively brief timeframe, are informative for policymakers, the availability of data prevents us from capturing the effects of ORDP on carbon emission over extended time horizons. Future studies may investigate the ORDP’s long-term effects on carbon emission to further comprehend the ramifications.

## Author contribution statement

DAN PAN: Conceived and designed the experiments; Analyzed and interpreted the data; Wrote the paper.

Yiqun Chen: Performed the experiments; Wrote the paper.

Fanbin Kong: Contributed reagents, materials, analysis tools or data; Wrote the paper.

## Funding statement

This study was supported by the National Natural Science Foundation of China (No. 71863016; No. 72063019); Humanities and Social Sciences Research Project of the Ministry of Education in China (No. 19YJCZH270).

## Data availability statement

Data will be made available on request.

## Declaration of interest’s statement

The authors declare no conflict of interest.

## Declaration of competing interest

The authors declare that they have no known competing financial interests or personal relationships that could have appeared to influence the work reported in this paper.
